# RT^2^ PCR array screening reveals distinct perturbations in DNA damage response signaling in FUS-associated motor neuron disease

**DOI:** 10.1186/s13041-019-0526-4

**Published:** 2019-12-04

**Authors:** Haibo Wang, Suganya Rangaswamy, Manohar Kodavati, Joy Mitra, Wenting Guo, Erika N. Guerrero, Ludo Van Den Bosch, Muralidhar L. Hegde

**Affiliations:** 1 Department of Radiation Oncology, Houston Methodist Research Institute, Houston, TX 77030 USA; 2 Center for Neuroregeneration, Department of Neurosurgery, Houston Methodist Neurological Institute, Houston Methodist Hospital, Houston, TX 77030 USA; 3 KU Leuven-Department of Neurosciences, Experimental Neurology and Leuven Brain Institute (LBI), 3000 Leuven, Belgium; 4 VIB, Center for Brain & Disease Research, Laboratory of Neurobiology, 3000 Leuven, Belgium; 5 Weill Medical College, New York, NY 10065 USA

**Keywords:** Motor neuron disease, Amyotrophic lateral sclerosis, DNA damage response, DNA repair, RT^2^ PCR array

## Abstract

Amyotrophic lateral sclerosis (ALS) is a degenerative motor neuron disease that has been linked to defective DNA repair. Many familial ALS patients harbor autosomal dominant mutations in the gene encoding the RNA/DNA binding protein ‘fused in sarcoma’ (FUS) commonly inducing its cytoplasmic mislocalization. Recent reports from our group and others demonstrate a role of FUS in maintaining genome integrity and the DNA damage response (DDR). FUS interacts with many DDR proteins and may regulate their recruitment at damage sites. Given the role of FUS in RNA transactions, here we explore whether FUS also regulates the expression of DDR factors. We performed RT^2^ PCR arrays for DNA repair and DDR signaling pathways in CRISPR/Cas9 FUS knockout (KO) and shRNA mediated FUS knockdown (KD) cells, which revealed significant (> 2-fold) downregulation of BRCA1, DNA ligase 4, MSH complex and RAD23B. Importantly, similar perturbations in these factors were also consistent in motor neurons differentiated from an ALS patient-derived induced pluripotent stem cell (iPSC) line with a FUS-P525L mutation, as well as in postmortem spinal cord tissue of sporadic ALS patients with FUS pathology. BRCA1 depletion has been linked to neuronal DNA double-strand breaks (DSBs) accumulation and cognitive defects. The ubiquitin receptor RAD23 functions both in nucleotide excision repair and proteasomal protein clearance pathway and is thus linked to neurodegeneration. Together, our study suggests that the FUS pathology perturbs DDR signaling via both its direct role and the effect on the expression of DDR genes. This underscors an intricate connections between FUS, genome instability, and neurodegeneration.

## Main text

The motor neuron disease amyotrophic lateral sclerosis (ALS) is characterized by the progressive degeneration of motor neurons in the motor cortex, brainstem and spinal cord with a life expectancy of 3–5 years after diagnosis. Ninety percent of ALS cases are sporadic disease with complex etiology, while about 10% are familial, a subset of which is caused by mutations in the gene encoding the RNA/DNA binding protein fused in sarcoma (FUS) [1–4]. Most of the over 50 mutations in FUS detected to date in ALS patients are clustered in or near its nuclear localization sequence (NLS), and thus induce cytoplasmic mislocalization [1]. FUS protein can bind directly to DNA contributing to D-loop formation and homologous DNA pairing [5]. FUS is recruited to laser-induced DNA damage tracks in a poly (ADP-ribose) polymerase 1 (PARP1)-dependent manner [6] and has been linked to DNA damage response (DDR) [7]. We recently demonstrated a role for FUS in DNA single-strand break (SSB) repair, where it regulates the recruitment and break-sealing function of XRCC1/DNA ligase 3 (LIG3) via direct interaction [8]. As an RNA binding protein, FUS regulates gene expression both at transcriptional and mRNA levels [1]. Although, its direct involvement in DNA repair and DDR signaling has received considerable attention, it is unclear whether FUS influences the expression of DDR factors.

**Fig. 1:**
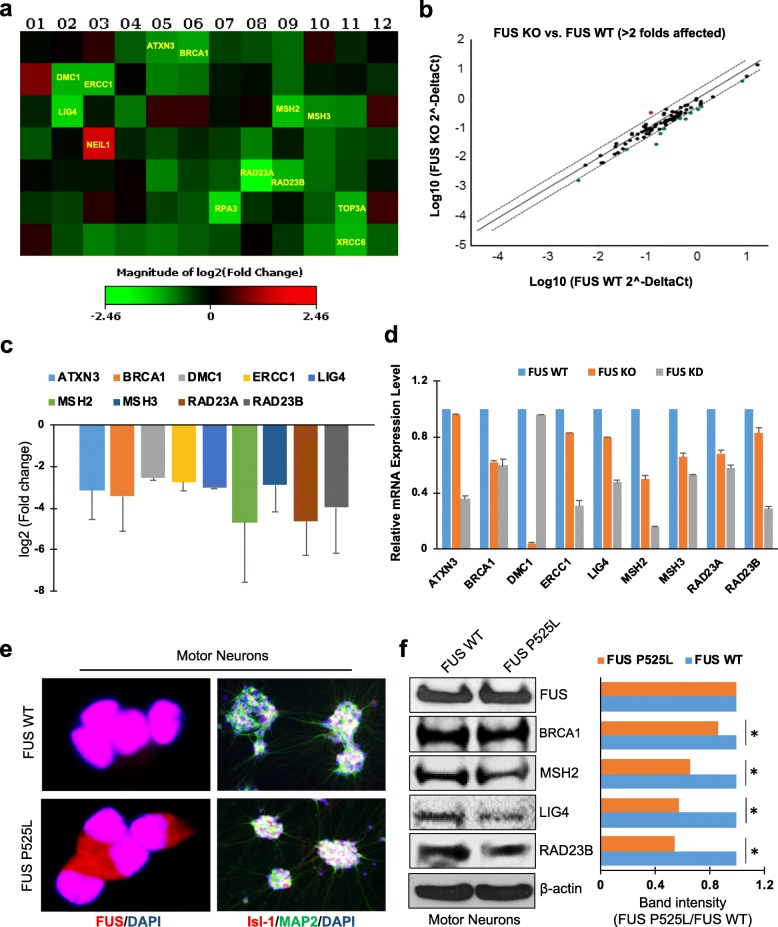
DNA repair and DDR gene expression profile by RT^2^ profiler PCR array in FUS knock out (KO) and knockdown (KD) cells, and its verification in ALS patient-derived motor neurons with a FUS P525L mutation reveal a complex perturbation pattern. **a** Heat map showing altered expressions of DNA repair genes in FUS KO cells. Red, green, and black squares indicate up-regulated genes, down-regulated genes, and non-regulated genes, respectively. **b** Scatter plot showing genes with > 2-fold difference in mRNA expression in FUS KO cells compared to control. Red, green, and black circles indicate up-regulated genes, down-regulated genes, and non-regulated genes, respectively. **c** Bar graph showing repair genes that were commonly down-regulated > 2 fold in FUS KO and KD cells compared to the control. **d** Histogram showing the relative mRNA expression level in FUS WT, FUS KO and FUS KD HEK293 cells. **e** Immunofluorescent labeling of motor neurons differentiated from ALS patient-derived iPSC lines for the indicated marker proteins. Representative images labeled for FUS shown cytoplasmic accumulation of FUS P525L mutant motor neurons. Labeled for Isl-1 and MAP 2 indicated ~ 80% differentiation efficiency of FUS WT and FUS P525L mutant iPSCs. Nuclei are stained with DAPI. **f** IB of endogenous FUS, BRCA1, MSH2, LIG4, and RAD23B in FUS WT and FUS P525L motor neurons. Histogram shows band intensity quantitation. *, *p* < 0.01. Error bars represent standard deviation from three independent experiments

To investigate whether loss of FUS affects expression of DDR factors, we first utilized a CRISPR/Cas9-mediated FUS knockout (KO) human embryonic kidney (HEK) 293 line [8]. The absence of FUS was confirmed by immunoblots of total extracts (Additional file 2: Figure S1). We then performed SYBR green-based quantitative real-time RT^2^ PCR arrays [9] for DDR (Qiagen, Cat#: PAHS-042Z). The 96-well RT^2^ profiler plate contained primers for 84 DDR genes (Additional file 1: Table S1), 5 housekeeping genes, and 3 negative control wells. The results revealed significant modulation (> 2-fold differential expression) in the expression of 13 DDR genes in FUS KO cells compared to control (Figs. 1a and b). We further performed similar RT^2^PCR profiler assay in shRNA-mediated FUS knockdown (KD) cells and identified 9 genes that are consistently downregulated (> 2-fold) in both FUS KO and KD cells. These are ataxin-3 (ATXN3, − 3.1-fold), breast cancer 1 (BRCA1, − 3.5-fold), disrupted meiotic cDNA1 (DMC1, − 2.5-fold), excision repair cross-complementing group 1 (ERCC1, − 2.73-fold), DNA ligase 4 (LIG4, − 3-fold), MutS homologs 2 (MSH2, − 4.7-fold) and 3 (MSH3, − 2.9-fold) and RAD23 homologs A (− 4.6-fold) and B (− 4-fold) (Fig. 1c). Overall, there were 3 upregulated genes and 42 downregulated genes in both FUS KO and KD cells (Additional file 1: Table S1).

To further confirm the down-regulation, we quantitated the mRNA level for the 9 genes in FUS KO and KD cells by performing quantitative (q) RT-PCR, and as shown in Fig.1d, the relative mRNA levels in FUS KO/KD cells were significantly down regulated compared with control cells, which is consistent with RT^2^ profiler assay. To attribute the reduced expression of the 9 genes to the nucleo-cytoplasmic mis-localization of FUS, we then tested the mRNA level of the 9 genes by qRT-PCR in fibroblast lines derived from normal person and a familial ALS patient carring FUS P525L mutation [10]. This mutation within the NLS of FUS induces nuclear clearance of FUS and is linked to a severe form of juvenile ALS [11]. As showed in Additional file 3 Figure S2, 8 of the 9 genes showed comparable pattern of reduced expression as in FUS KO and KD cells, while the mRNA level of BRCA1 in FUS mutated cells is moderately up regulated compared with FUS WT cells.

Next, we validated the differential expression of DDR factors in motor neurons differentiated from induced pluripotent stem cells (iPSCs) derived from the fibroblasts by immunoblotting (IB). iPSCs were differentiated into motor neurons as we described previously [8, 10], and immunofluorescence labeling with the neuronal marker MAP 2 and the motor neuron-specific marker Isl-1 confirmed effective differentiation (Fig. 1e). As expected, the FUS P525L displayed cytoplasmic accumulation compared with FUS WT (Fig. 1e). IB of total cell extracts from FUS WT and FUS P525L motor neurons confirmed the downregulation of MSH2, LIG4, and RAD23B in FUS P525L mutant motor neurons (Fig. 1f). Interestingly, although the mRNA level of BRCA1 in FUS P525L fibroblasts is comparable with FUS WT, its protein level is slightly reduced in FUS mutated cells (Fig. 1f). Similar pattern was seen in antisense oligonucleotide-mediated FUS KD motor neurons (Additional file 4: Figure S3a). We also observed down regulated expression level of BRCA1 and MSH2 in human postmortem spinal cord tissue from two sporadic ALS patients with FUS pathology (Additional file 4: Figure S3b). Control and ALS tissue were obtained as de-identified specimens from the Department of Veterans Affairs Brain Biorepository, USA. Cytoplasmic mis-localization of FUS was previously demonstrated in these patient specimens [8]. Finally, GeneMANIA pathway analysis software [12] was used to predict gene-gene functional interactions, which revealed connections between FUS and many of the DDR factors identified in this study, thus consistent with our experimental results (Additional file 5: Figure S4).

DNA repair and an effective DDR are critical for maintaining the integrity of the genome, and deficiency and altered signaling have been linked to neurodegenerative disorders including ALS. Here, we identified dysregulation of a number of canonical DDR genes in human cells that lack FUS or ALS patient-derived motor neurons that express the familial P525L mutation in FUS. It is important to note that 9 genes that were > 2-fold downregulated in KO cells, were consistently downregulated in patient-derived mutant cells, except for BRCA1. This suggest that in terms of their role in regulating DDR factors’ gene expression, this ALS-linked mutation FUS-P525L behaved as “loss-of-normal FUS function”.

The similar DDR perturbations in ALS patient spinal cord specimens corroborate the important RNA processing role of FUS in regulating DDR and link FUS pathology to altered DDR signaling in ALS. Downregulation of the DNA mismatch repair (MMR)-associated MSH2/MSH3 complex could inhibit repair of base-base mismatches that occur during repair synthesis or replication of GT mismatches normally caused by deamination of G:5-MethylC. MSH complexes also repair small looped DNA lesions composed of 2–13 bases, and MMR defects may be a major source of insertion-deletion mutations in ALS [14]. LIG4 is a nuclear enzyme that joins DNA double-strand breaks (DSBs) via non-homologous end joining (NHEJ) repair [13]. This, together with our recent report of inhibition of LIG3 function and SSB repair in FUS-associated ALS [8], suggests perturbations in both SSB and DSB repair in ALS.

Notably, in addition to their canonical role in DNA repair, some DDR factors possess secondary noncanonical function(s), whose defects may also affect neurodegeneration. For example, RAD23 is involved in nucleotide excision repair (NER) as a damage sensor [13] and also functions in the proteasomal pathway as an ubiquitin receptor and is linked to toxic protein clearance machinery in neurodegenerative disease [14]. The tumor suppressor BRCA1 plays a key role in DSB repair via homologous recombination (HR) pathway, and BRCA1 KD mice accumulate DSBs in neuronal genomes, although the role of HR in postmitotic neurons is unclear [13]. Notably, while both BRCA1 mRNA and proteins levels were consistently down regulated in most cell types, including motor neurons derived from ALS patient with FUS P525L mutation and ALS patient spinal cord tissues with FUS pathology, redcued BRCA1 protein levels was not consistent with mRNA level in the patient fibroblasts, likely due to unexplored regulation at the protein level, which needs further investigation. Interestingly, reduced BRCA1 expression in brain tissue from patients with Alzheimer disease is correlated to cognitive and learning defects [15]. Thus, FUS pathology-mediated loss of BRCA1 may contribute to cognitive defects, particularly in FUS-associated frontotemporal dementia (FTD).

In conclusion, this report reveals a complex pattern of perturbations in canonical DNA repair and DDR pathways in FUS-associated neurodegeneration involving both its direct role in DDR and gene regulatory function. Inhibition of the noncanonical roles of DDR factors may contribute to FUS related neurodegeneration.

## Supplementary information

**Additional file 1: Table S1.** List of DNA repair genes screened by RT2 profiler array in FUS knockout and knockdown cell. The histogram shows the number of total genes and genes that are consistently down regulated or up regulated in both FUS KO and KD cells.

**Additional file 2: Figure S1.** Immunoblot (IB) showing FUS KO by CRISPR/Cas9 in HEK293 cells. β-actin was probed as a loading control.

**Additional file 3: Figure S2.** Histogram showing the relative mRNA expression level of altered DNA repair genes in FUS WT and FUS P525L fibroblasts.

**Additional file 4: Figure S3.** Validation of protein levels of DDR factors in FUS KD motor neurons and ALS spinal cord with FUS pathology.

**Additional file 5: Figure S4.** Gene-gene functional interaction network prediction using GeneMANIA pathway analysis software.

## Data Availability

All data generated or analyzed during this study are included in this published article.

## Abbreviations

ALS: Amyotrophic lateral sclerosis
ATXN3: Ataxin-3
BER: Base excision repair
BRCA1: Breast cancer 1
DDR: DNA damage response
DMC1: Disrupted meiotic cDNA1
DSB: Double-strand break
ERCC1: Excision repair cross-complementing group 1
HR: Homologous recombination
iPSCs: Induced pluripotent stem cells
LIG4: DNA ligase 4
MMR: Mismatch repair
MSH: MutS homolog
NEIL1: Nei-like DNA glycosylase 1
NHEJ: Non-homologous end joining
NLS: Nuclear localization sequence
RAD23A: RAD23 homologs A
RAD23B: RAD23 homologs
SSB: Single-strand break
