# Supplementary material for: RT2 PCR array screening reveals distinct perturbations in DNA damage response signaling in FUS-associated motor neuron disease
Source: Mol Brain. 2019 Dec 4;12:103. doi: 10.1186/s13041-019-0526-4 (PMC6894127; doi:10.1186/s13041-019-0526-4)
Supplement: Supplementary file 1 — Additional file 1: Table S1. List of DNA repair genes screened by RT2 profiler array in FUS knockout and knockdown cell. The histogram shows the number of total genes and genes that are consistently down regulated or up regulated in both FUS KO and KD cells. [file 13041_2019_526_MOESM1_ESM.pdf]

RT^2 profiler whole gene list					
APEX1	ERCC1	MLH3	OGG1	RAD23B	TOP3A
APEX2	ERCC2	MMS19	PARP1	RAD50	TOP3B
ATM	ERCC3	MPG	PARP2	RAD51	TREX1
ATR	ERCC4	MRE11A	PARP3	RAD51B	UNG
ATXN3	ERCC5	MSH2	PMS1	RAD51C	XAB2
BRCA1	ERCC6	MSH3	PMS2	RAD51D	XPA
BRCA2	ERCC8	MSH4	PNKP	RAD52	XPC
BRIP1	EXO1	MSH5	POLB	RAD54L	XRCC1
CCNH	FEN1	MSH6	POLD3	RFC1	XRCC2
CCNO	LIG1	MUTYH	POLL	RPA1	XRCC3
CDK7	LIG3	NEIL1	PRKDC	RPA3	XRCC4
DDB1	LIG4	NEIL2	RAD18	SLK	XRCC5
DDB2	MGMT	NEIL3	RAD21	SMUG1	XRCC6
DMC1	MLH1	NTHL1	RAD23A	TDG	XRCC6BP1

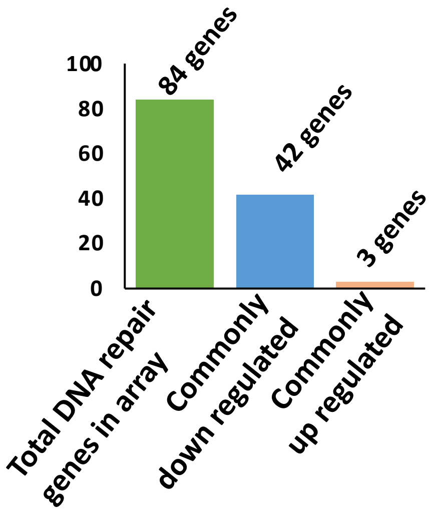

Additional file: Table S1. List of DNA repair genes screened by RT^2 profiler array in FUS knockout and knockdown cell. The histogram shows the number of total genes and genes that are consistently down regulated or up regulated in both FUS KO and KD cells.
