# Supplementary material for: RT2 PCR array screening reveals distinct perturbations in DNA damage response signaling in FUS-associated motor neuron disease
Source: Mol Brain. 2019 Dec 4;12:103. doi: 10.1186/s13041-019-0526-4 (PMC6894127; doi:10.1186/s13041-019-0526-4)
Supplement: Supplementary file 2 — Additional file 2: Figure S1. Immunoblot (IB) showing FUS KO by CRISPR/Cas9 in HEK293 cells. β-actin was probed as a loading control. [file 13041_2019_526_MOESM2_ESM.pdf]

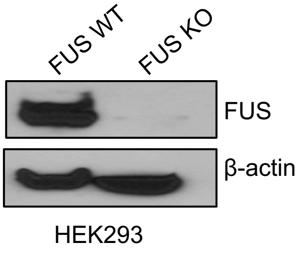

Additional file: Figure S1. Immunoblot (IB) showing FUS KO by CRISPR/Cas9 in HEK293 cells. β-actin was probed as a loading control.
