# Supplementary material for: RT2 PCR array screening reveals distinct perturbations in DNA damage response signaling in FUS-associated motor neuron disease
Source: Mol Brain. 2019 Dec 4;12:103. doi: 10.1186/s13041-019-0526-4 (PMC6894127; doi:10.1186/s13041-019-0526-4)
Supplement: Supplementary file 3 — Additional file 3: Figure S2. Histogram showing the relative mRNA expression level of altered DNA repair genes in FUS WT and FUS P525L fibroblasts. [file 13041_2019_526_MOESM3_ESM.pdf]

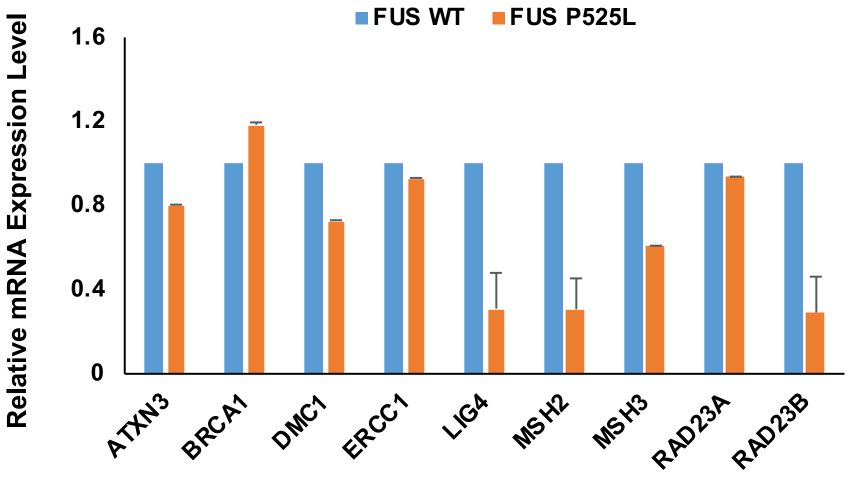

Additional file: Figure S2. Histogram showing the relative mRNA expression level of altered DNA repair genes in FUS WT and FUS P525L fibroblasts.
