# Supplementary material for: RT2 PCR array screening reveals distinct perturbations in DNA damage response signaling in FUS-associated motor neuron disease
Source: Mol Brain. 2019 Dec 4;12:103. doi: 10.1186/s13041-019-0526-4 (PMC6894127; doi:10.1186/s13041-019-0526-4)
Supplement: Supplementary file 4 — Additional file 4: Figure S3. Validation of protein levels of DDR factors in FUS KD motor neurons and ALS spinal cord with FUS pathology. [file 13041_2019_526_MOESM4_ESM.pdf]

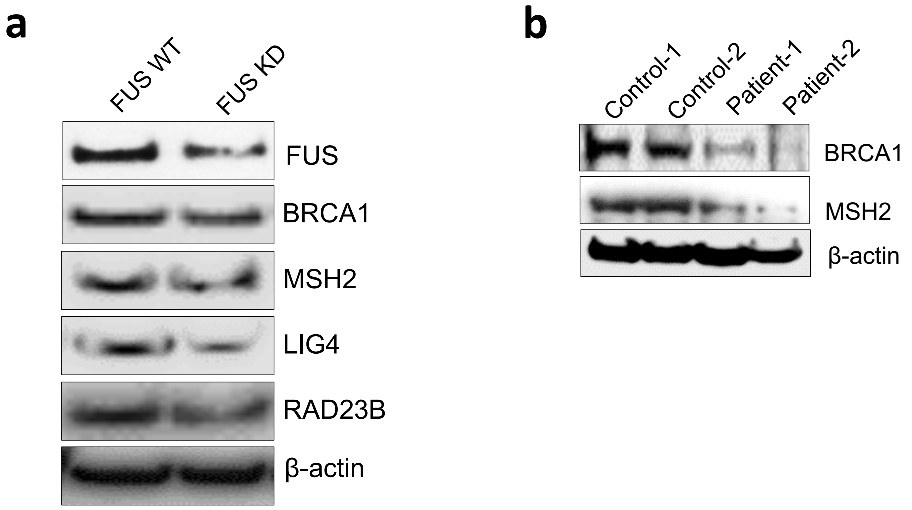

Additional file: Figure S3. Validation of protein levels of DDR factors in FUS KD motor neurons and ALS spinal cord with FUS pathology. (**a**) IB of endogenous BRCA1, MSH2, LIG4 and RAD23B in FUS WT antisense oligonucleotide-mediated FUS KD motor neurons. (**b**) IB of endogenous BRCA1 and MSH2 in control and ALS spinal cord tissue.
